# Primary rare anaplastic large cell lymphoma, ALK positive in small intestine: case report and review of the literature

**DOI:** 10.1186/s13000-016-0539-6

**Published:** 2016-09-09

**Authors:** Qinghua Cao, Fang Liu, Shurong Li, Ni Liu, Lihui Li, Changzhao Li, Tingsheng Peng

**Affiliations:** 1 Department of Pathology, The First Affiliated Hospital of Sun Yat-sen University, #58, Zhongshan Road II, Guangzhou, 510080 China; 2 Department of Oncology, Nanfang Hospital, Southern Medical University, #1838 Guangzhou Dadao Bei Avenue, Guangzhou, 510515 China; 3 Department of Radiology, The First Affiliated Hospital of Sun Yat-sen University, Guangzhou, 510080 China

**Keywords:** Anaplastic large cell lymphoma, ALK positive, Small intestine

## Abstract

**Background:**

Primary anaplastic large cell lymphoma, ALK positive in small intestine is clinically rare ﻿and the clinical, radiological and pathological information are generally not ﻿availab﻿le﻿. Here, we report a case of 32-year-old male with ALK positive anaplastic large cell lymphoma at the junction of jejunum and ileum, and highlight the clinicopathological features and the differential diagnosis of this type lymphoma.

**Case presentation:**

The patient presented with right middle abdominal mass for 1 month with sporadic pain. Computed tomography (CT) showed a mass measured 8.5 × 7.4 × 4 cm at the junction of jejunum and ileum. The diagnosis was made after pathological examination of the excised tissue by enterectomy. Grossly, the mass was located predominately in intestinal wall with grayish appearance and blurry boundary. Microscopically, almost all layers of the intestinal wall were infiltrated by pleomorphic tumor cells with diffuse and cohesive growth pattern. The neoplastic cells were mainly medium to large size with moderate basophilic cytoplasm. Most of them had hyperchromatic nuclei and prominent nucleoli. “Hallmark” cells were easily detected. Immunohistochemically, tumor cells are characterized by CD30, ALK, CD5, TIA-1, Granzyme B, EMA positive staining, and CD2, CD3, CD7, CD4, CD8, CD20, CD79a negative staining. The Epstein-Barr virus encoded RNAs (EBERs) genome was also negative. A diagnosis as primary small intestinal ALK positive anaplastic large cell lymphoma was finally made. The patient received CHOP chemotherapy and is alive till now without recurrence 5 months after enterectomy.

**Conclusions:**

Primary small intestinal ALK positive anaplastic large cell lymphoma is rare. The accurate diagnosis should be based on combined consideration of clinical characteristics, CT image and pathological features, and should be distinguished from other lymphomas or solid tumors in small intestine.

## Background

Anaplastic large cell lymphoma (ALCL) was first identified by Stein et al. in 1985 [1]. It belongs to peripheral T-cell lymphoma characterized by large neoplastic cells with abundant cytoplasm and eccentric horseshoe- or kidney-shaped nuclei. According to the WHO classification (4^th^ Edition), primary systemic ALCL includes two distinct entities: anaplastic lymphoma kinase (ALK) positive and ALK negative [2]. ALCL mostly affects lymph nodes, with uncommon involvement of extra-nodal sites including soft tissue, bone, lung and liver [3]. Cases of ALCL involving small intestine are therefore very rare. So far, there are only a few case reports in the literature [4–7]. Nevertheless, detailed clinical, radiological and/or pathological information of these cases are generally not available. Herein, we report a rare case of primary ALK positive ALCL involving small intestine accompanied with comprehensive clinicopathological features and discussion of differential diagnosis.

## Case presentation

### Clinical history

**Fig. 1:**
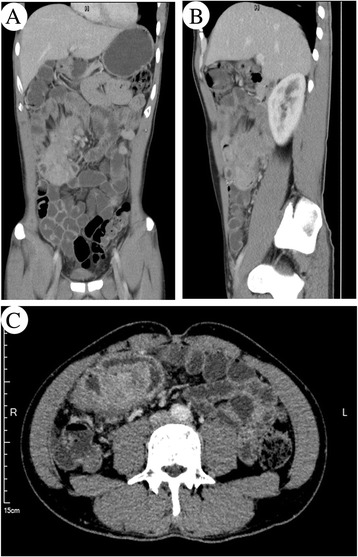
Computed tomography (CT) scan of an abdominal mass. CT showed a mass involved small intestines measured 8.5 × 7.4 × 4 cm in coronal view (**a**) sagittal view (**b**) and transverse section (**c**)

A 32-year-old man presented with right middle abdominal mass for 1 month and sporadic pain without fever, nausea, vomit or diarrhea. The patient was admitted to our hospital due to sudden severe pain in the abdomen. Physical examinations showed no abnormality and there was absence of palpable superficial lymphadenopathy. A computed tomography (CT) scan showed a mass measured 8.5 × 7.4 × 4 cm at the junction of jejunum and ileum involving the adjacent intestinal wall and adipose tissue (Fig. 1). The colon and rectum were not affected, and there was no obvious involvement of the mesenteric lymph nodes in the immediate vicinity. The liver and spleen were also normal in CT image. There was no obvious enlargement of the mediastinal lymph nodes by CT scanning of thorax. Laboratory examinations revealed negative results for tumor markers of digestive tract including AFP, CEA, CA125, SCC and CA199. Blood routine examination showed white blood cell count at 5.42 × 10^9^/L, the lymphocyte count at 1.5 × 10^9^/L, the red cell count at 5.33 × 10^12^/L, hemoglobin count at 142 g/L, and the platelet count at 350 × 10^9^/L respectively. All the parameters of the peripheral blood cells were found in normal range. After the final diagnosis as primary ALK positive anaplastic large cell lymphoma, the patient received CHOP chemotherapy and is alive till now without recurrence 5 months after jejunectomy and ileectomy. Material and methods‚ The specimen was fixed in a 10 % neutral formalin solution and embedded in paraffin. A 4-μm-thick section was stained with hematoxylin-eosin (H&E) for routine microscopy. Immunohistochemical (IHC) staining was performed using the EnVision Detection System (DAKO, Denmark). Commercially available monoclonal antibodies were employed CD20 (Mouse mAb(L26);1:200), CD79a (Mouse mAb;1:200), CD2 (Mouse mAb(AB75);1:200), CD3ε (Mouse mAb (F7.2.38);1:200), CD5 (Mouse mAb(CD5/54/F6);1:300), CD7 (Mouse mAb (CBC. 37);1:200), CD4 (Mouse mAb(4B12);1:200), CD8 (Mouse mAb (C8/ 144B);1:200),CD30 (Mouse mAb(Ber-H2);1:200), ALK (Mouse mAb (ALK1);1:200), Granzyme B (Mouse mAb(GrB-7);1:400), EMA (Mouse mAb(MC-7);1:200), CD10 (Mouse mAb(SS2/36);1:200), Bcl-2 (Mouse mAb(8C8); 1:500), Bcl-6 (Mouse mAb (PG-B6p);1:200), MUM-1(Mouse mAb(Mumlp);1:200), CD38(Mouse mAb (HIT2);1:200), CD138(Mouse mAb(MI15);1:200), CD56(Mouse mAb(123C3);1:300), CyclinD1 (Mouse mAb(DCS-6);1:200), CD21 (Mouse mAb(1 F8);1:200), Kappa (Mouse mAb(R10-21-F3);1:400), Lambda (Mouse mAb(N10/2);1:200) and Ki67 (Mouse mAb(MIB-1);1:200). The primary antibodies of TIA-1(Mouse mAb (2G9A10F5);1:200) were from Zhongshan company.

In situ hybridization (ISH) with Epstein-Barr virus (EBV)-encoded small RNA (EBER) oligonucleotides was used to test the specimens for the presence of EBV small RNA in formalin-fixed, paraffin-embedded sections using a hybridization kit (DAKO, Denmark).

### Pathological findings

**Fig. 2:**
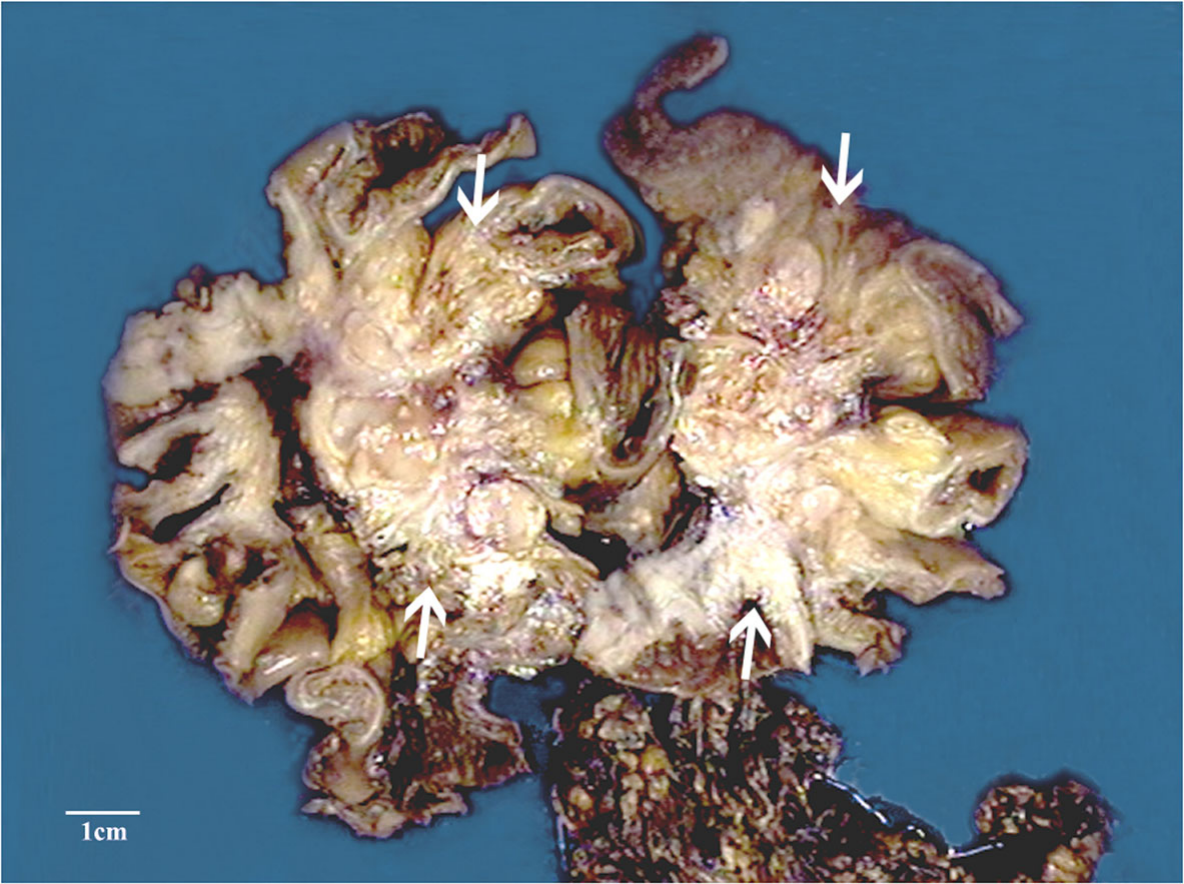
Macroscopic appearance of the intestinal mass (arrows). Cut surface showed normal structures of intestinal wall were totally destroyed. The mass was located predominately in intestinal wall with grayish appearance and breezing boundary

Grossly, the mass is measured as 9 × 7.5 × 4 cm with local invasion of small intestine. On the cut surface, normal structures of intestinal wall were totally destroyed. The mass was located predominately in intestinal wall with grayish appearance and unclear boundary (Fig. 2). Hemorrhage and necrosis were absent.

**Fig. 3:**
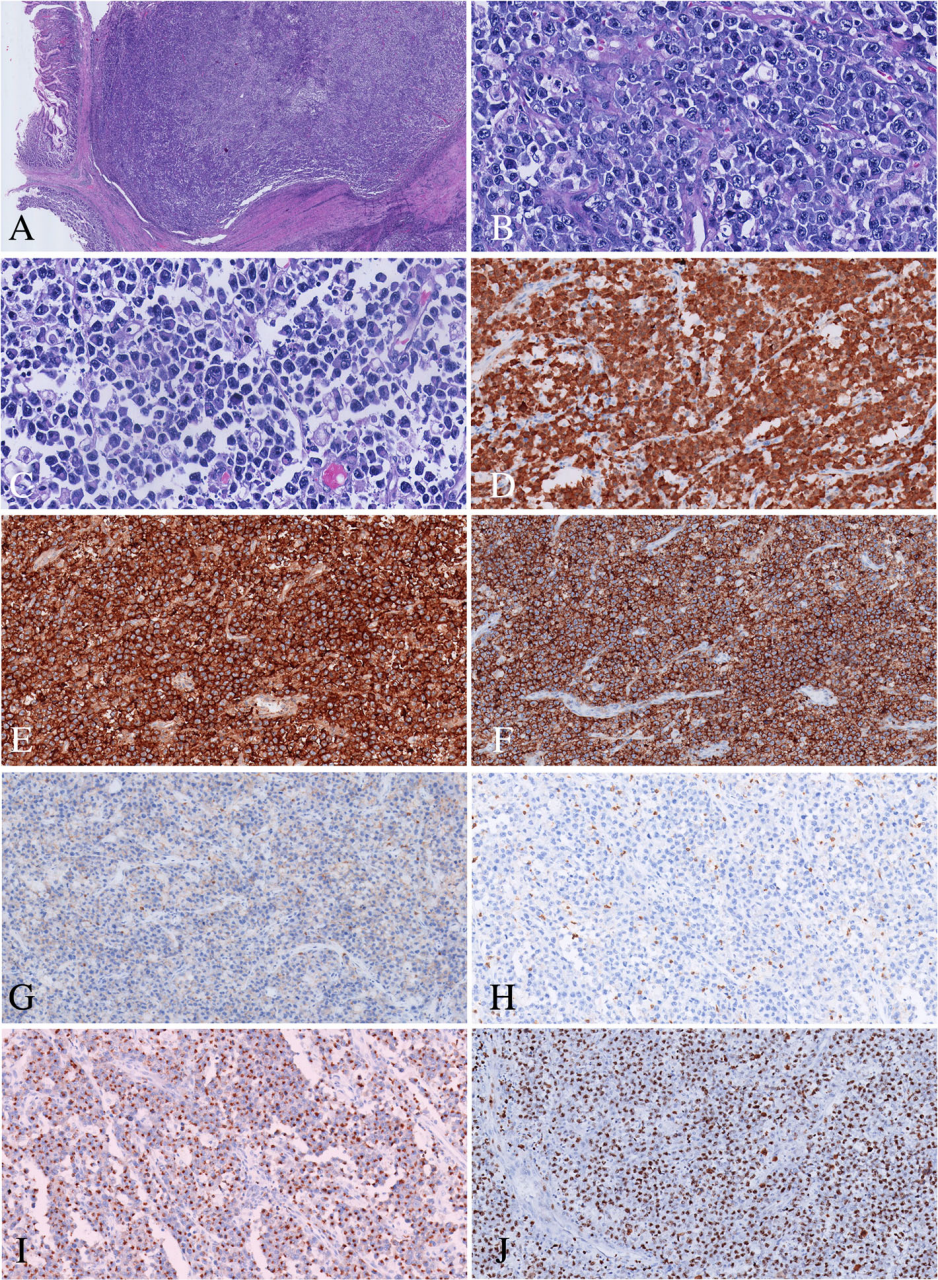
Microscopic features of the intestinal tumor. **a** The intestinal wall were infiltrated by pleomorphic tumor cells with sheet-like pattern (HE × 40); **b c** The neoplastic cells were mainly medium to large size with moderate pale cytoplasm, hyperchromatic nuclei and prominent nucleoli. “Hallmark” cells were easily found. Reed-Sternberg cell-like cell can be detected (HE × 400); **d** Tumor cell showed nuclear and cytoplasmic staining for ALK; Tumor cells were positive for CD30 **e** CD5 **f** TIA-1 **i** negative for CD4 **g** CD8 **h** (Envision × 100); **j** Ki67 staining showed almost 80 % proliferation index (Envision × 100)

Microscopically, almost all layers of the intestinal wall were infiltrated by pleomorphic tumor cells with diffuse and cohesive proliferation pattern (Fig. 3a). The neoplastic cells were mainly medium to large size with moderate slightly basophilic cytoplasm. Most of them had hyperchromatic nuclei and prominent nucleoli showing immunoblastic and plasmablastic cell features. Hallmark large cells with eccentric horseshoe- or kidney-shaped nuclei often with an eosinophilic region near the nucleus, were detected easily. Additionally, neoplastic cells with mirror-image nuclei resembling Reed-Sternberg cells were also identified (Fig. 3b, c). Mitotic cells were frequently observed.

Immunohistochemically, the neoplastic cells were ALK positive with both nuclear and cytoplasm staining (Fig. 3d). In addition, these cells are positive for CD30 (Fig. 3e), CD5 (Fig. 3f), TIA-1 (Fig. 3i), Granzyme B and EMA, whereas negative for all of others biomarkers including CK, CD2, CD3, CD7, CD4 (Fig. 3g), CD8 (Fig. 3h), CD56, CD10, Bcl-6, Mum-1, Bcl-2, CD38, CD138, Kappa, Lambda, CD21, and CyclinD1. The proliferation index was approximately 80 % as assessed by Ki-67 staining (Fig. 3j). In situ hybridization (ISH) for EBERs study showed no positive signals.

Based on the above findings, this tumor was diagnosed as anaplastic large cell lymphoma (ALCL), ALK positive. Bone marrow biopsy revealed no tumor cells infiltration.

## Discussion

Primary gastrointestinal (GI) tract lymphoma is uncommon and only accounts for 1–4 % of all GI malignances [8]. According to Dawson et al. [9], the diagnosis of GI lymphoma should meet the following 5 criteria: (1) absence of palpable superficial lymphadenopathy; (2) no evidence of enlarged mediastinal lymph nodes; (3) normal total and differential white blood cell counts; (4) a predominance of bowel lesions at laparotomy with only the lymph nodes obviously affected in the immediate vicinity; (5) absence of tumor involvement of the liver and spleen. Small intestine is the second most common site affected by primary GI lymphoma which ranks after stomach [10]. Intestinal B-cell lymphomas occur more frequently than T-cell lymphomas at the ratio of 6:1 [11]. As reported, the most common subtype of primary intestinal lymphoma is diffuse large B-cell lymphoma and extra-nodal marginal zone lymphoma of mucosa-associated lymphoid tissue (MALT lymphoma) [10–12]. Among the T-cell lymphomas of GI tract, peripheral T-cell lymphomas (PTCL), enteropathy-associated T-cell lymphoma (EATL) and NK/T-cell lymphoma (NK/TL) are the common subtypes [12, 13]. In a retrospective Korean study, 42 primary GI T-cell lymphomas were assessed out of which 17 cases are located in small intestine. Among the 17 small intestinal T-cell lymphomas, 10 lesions (58.8 %) could be classified as EATL type II, 4 cases (23.5 %) as NK/TL, 2 cases (11.8 %) as PTCL, and only 1 case (9.5 %) as ALCL [5]. Another study from Japan reported only 1 case of ALCL (6.7 %) among 15 intestinal T-cell lymphomas in small intestine [6]. Hence, ALCL primarily occurred from small intestine is very rare.

According to WHO classification of tumors of hematopoietic and lymphoid tissues (4th Edition), ALCL was divided into ALK-positive and ALK-negative subtypes based on expression of ALK or ALK gene rearrangement [2]. ALK/Nucleophosmin (NPM1) gene fusion was the most common genetic alteration. In addition, variant ALK rearrangement partners have been recognized subsequently including TPM3, ATIC, and TFG. Mechanistically, rearrangements of these genes result in the up-regulation of ALK protein. Moreover, the pattern of ALK immunostaining depends on different types of genetic alterations [14, 15]. In this regard, ALK/NPM1 fusion is characterized by both nucleus and cytoplasm staining, whereas fusions of ALK with other partners lead to diffuse cytoplasmic staining only [16]. Compared with ALK-negative ALCL, ALK positive ALCL occurs more frequently in younger patients with favorable outcomes [17, 18]. Most of the patients diagnosed with ALK negative ALCL are reported to have cutaneous, hepatic, or gastrointestinal lesions [18]. In this regard, Kim identified 4 ALK negative ALCL in GI tract including 2 in duodenum, 1 in large intestine, and 1 in small intestine [5]. In another report by Carey MJ, only 3 ALK negative cases were found out of 4 small intestinal ALCL [4]. Based on our observations and other reports, it seems that ALK negative ALCL tends to invade intestine. However, since the number of cases is very small, a larger patient cohort is needed to make a solid conclusion.

Moreover, diagnosis of intestinal ALCL should be made after exclusion of other lymphomas which show similar cell morphology and/or ALK positive staining. The most important differential diagnosis was ALK positive diffuse large B cell lymphoma (ALK positive DLBCL), which was now considered as a distinct entity [2, 19]. It was characterized by monomorphic large plasmablast- or immunoblast-like cells with large central nucleoli. It is histologically very close to ALCL and metastatic carcinoma in lymph node at low magnification because of its sinusoidal growth pattern. The neoplastic cells often lack the expression of lineage-associated leukocyte antigens as CD3, CD5, CD20, or CD79a, but express ALK [2, 19, 20]. Therefore it could lead to the diagnosis of ALCL “null cell” phenotype. However, ALK positive DLBCL constantly lack the expression of CD30 in immunohistochemistry, and strongly express plasma cell markers such as CD138 and CD38, which demonstrates the features of terminally differentiated B-lineage origin of the tumor cells. Moreover, unlike both the nucleus and cytoplasm staining in ALCL, the staining of ALK in DLBCL exhibits granular cytoplasmic staining indicating the CLTC/ALK fusion protein [19, 20]. In the present case, besides ALK and CD30 expression, the neoplastic cells were also positive for T lymphocyte lineage markers as CD5, TIA-1, and Granzyme B, while negative for CD20, CD79a, CD38, CD138, suggesting the T-cell origin of these cells and the diagnosis of ALK positive ALCL.

Plasmablastic Lymphoma (PBL) should also be considered as one of the differential diagnosis because of the morphology of the neoplastic cells mimicking centroblastic and immunoblastic cells. The neoplastic cells of plasmablastic lymphoma expressed markers characterizing plasma cell phenotype including CD 138, CD38, Vs38c either kappa or lambda light chain, and IRF4/MUM1, whereas without the expression of T cell phenotype. Although the neoplastic cells frequently expressed EMA or CD30, they did not express ALK [2, 21]. Moreover, EBV EBER in situ hybridization is positive in 60–75 % of the cases of PBL [2, 21]. Based on the immunophenotype in this case, the diagnosis as PBL was not correct.

Peripheral T-cell lymphoma, not other specified (PTCL, NOS) deserves additional attention in terms of differential diagnosis. It encompassed all mature T-cell neoplasms lacking specific features that would allow its categorization in any of the better-defined subtypes of post-thymic T-cell lymphoma. In some cases, tumor cells infiltrate lymphatic sinuses and consist of CD30 positive large cells predominantly, which result in difficulty of distinguishing it from ALK negative ALCL. In fact, diagnostic criteria for ALK negative ALCL are still evolving, particularly in distinguishing it from other CD30 positive PTCL, NOS [15, 22]. In ALK negative ALCL, all tumor cells are positive for CD30, mainly on the cell membrane and in the Golgi region. Particularly, the staining of CD30 are strong and of equal intensity in all the tumor cells [16, 23]. By contrast, CD30 staining in PTCL, NOS is usually heterogeneous and comparatively weaker than that in ALK negative ALCL [23]. Additionally, the diagnosis of ALK negative ALCL should be reserved for cases with the morphology of ALCL accompanied with a cytotoxic T-cell phenotype including TIA-1, perforin and Granzyme B [24]. Nevertheless, other pathologists consider ALK negative ALCL as an anaplastic variant of PTCL, NOS, in light of its poor prognosis and partially overlapping phenotype [18, 25, 26].

In addition, ALCL occured in the GI tract must be distinguished from enteropathy associated T-cell lymphoma (EATL), type I, which is considered as a complication of celiac disease (gluten-sensitive). In Type I EATL, the cytological composition of tumor cells is varied, and more polymorphous than Type II lymphomas [25, 27]. In this regard, ALK negative ALCL may closely resemble CD30 positive EATL. However, unlike ALCL, usually EATL is not positive for both EMA and ALK. In addition, EATL is mainly CD8 positive rather than CD4 positive. Its association with celiac disease or villous atrophy further distinguishes itself with ALK positive ALCL [2, 28].

Since some of the tumor cells in ALCL resemble classic Reed-Sternberg cells which are “hallmark” cells in Hodgkin’s lymphoma (HL), differential diagnosis should also include HL. Fortunately, it is not difficult to differentiate HL from ALCL if it is ALK positive, but at times it may be a challenge when it comes to ALK negative ALCL. In this case, expression of EMA, clusterin, or CD56 in tumor cells may help to support a diagnosis of ALK negative ALCL, as the staining of these markers are known to be absent in HL [29]. Jaffe have suggested that HL-like ALK negative ALCL should be diagnosed only when tumor cell morphology closely resemble Hodgkin’s lymphoma but immunohistochemical study show characteristics of ALCL (positive staining for CD30, EMA, CD3 but negative for EBV and B-cell markers) [24].

In addition to lymphatic tissue tumor, ALK rearrangements can also be detected in other malignancies, including inflammatory myofibroblastic tumors [30, 31], non-small cell lung cancers [32], neuroblastomas [33] and embryonal rhabdomyosarcomas [34]. However, these malignancies are readily distinguishable from ALCL is based on their distinct morphology. Moreover, some specific immunohistochemistry markers which were not known to be expressed in ALCL but in the other malignancies can support the diagnosis, including rhabdomyosarcoma markers MyoD1 and Myogenine and non-small cell lung cancers marker TTF-1.

In general, we reported a rare case of primary intestinal ALK positive large cell lymphoma and highlighted the clinicopathological features and differential diagnosis.

## Conclusions

The case reported a rare primary anaplastic large cell lymphoma in small intestine. As intestine can be involved by other types of lymphomas, the final diagnosis should be made with the combination of clinical feature, PET-CT image, pathological morphology, immunophenotype, and genetic features. For this patient, the final diagnosis was made as primary ALCL, ALK positive, stage IIIA. Although the prognosis depends on the response to chemotherapy, ALK positive may predicts a favorable prognosis in this case.

## Abbreviations

ALCL: Anaplastic large cell lymphoma
CT: Computed tomography
DLBCL: Diffuse large B cell lymphoma
EATL: Enteropathy-associated T-cell lymphoma
EBER: Epstein-Barr virus (EBV)-encoded small RNA
GI: Gastrointestinal
HL: Hodgkin’s lymphoma
IHC: Immunohistochemistry
ISH: In situ hybridization
MALT: Extranodal marginal zone lymphoma of mucosa-associated lymphoid tissue
NK/TL: NK/T-cell lymphoma
PBL: Plasmablastic Lymphoma
PTCL: NOS, peripheral T-cell lymphoma, not other specified
